# Effects of active breaks on educational achievement in children with and without ADHD: study protocol and rationale of the Break4Brain project

**DOI:** 10.3389/fpsyg.2024.1451731

**Published:** 2024-10-31

**Authors:** Diego Arenas, Miranda Bodi-Torralba, Andrea Oliver, Jaume Cantallops, Francisco J. Ponseti, Pere Palou-Sampol, Juan A. Collado, Isabel Flórez, Alejandro Galvez-Pol, Juan L. Terrasa, Carolina Sitges, Víctor Sánchez-Azanza, Raúl López-Penadés, Daniel Adrover-Roig, Adrià Muntaner-Mas

**Affiliations:** ^1^GICAFE “Physical Activity and Exercise Sciences Research Group”, Faculty of Education, University of the Balearic Islands, Palma, Spain; ^2^Department of Pedagogy and Specific Didactics, Institute of Research and Innovation in Education, University of the Balearic Islands, Palma, Spain; ^3^Department of Education, Valencian International University, Valencia, Spain; ^4^Balearic Institute of Mental Health of Children and Adolescents (IBSMIA), Son Espases University Hospital, Health Research Institute of the Balearic Islands (IdISBa), Palma, Spain; ^5^Psychology Department, University of the Balearic Islands, Palma de Mallorca, Palma, Spain; ^6^Active Cognition, Embodiment, and Environment Lab, University of the Balearic Islands, Palma de Mallorca, Palma, Spain; ^7^Research Institute of Health Sciences (IUNICS), Health Research Institute of the Balearic Islands (IdISBa), Palma, Spain; ^8^Department of Psychology, University of the Balearic Islands (UIB), Palma, Spain; ^9^Department of Applied Pedagogy and Educational Psychology, Institute of Research and Innovation in Education (IRIE), University of the Balearic Islands, Palma, Spain; ^10^PROFITH “PROmoting FITness and Health Through Physical Activity” Research Group, Sport and Health University Research Institute (iMUDS), Faculty of Sport Sciences, University of Granada, Granada, Spain

**Keywords:** cross-over, cluster randomized controlled trial, physically active lessons, active classroom, EEG, cognition, academic performance, school

## Abstract

The Break4Brain project aims to elucidate the effects of both acute and chronic physical activity (PA) on educational achievement in children with and without Attention Deficit Hyperactivity Disorder (ADHD). This study will be conducted in two phases: a cross-over design followed by a hybrid type 1 implementation-effectiveness trial, which includes both a cluster randomized controlled trial (RCT) and a qualitative study. In phase I, 60 children aged 10–12, with 30 each from ADHD and non-ADHD groups, will participate in a laboratory-based study over 4 days within 1 month. They will participate in three counterbalanced experimental conditions: (i) PA with cognitive engagement, (ii) PA without cognitive engagement, and (iii) a cognitively engaging control. This phase will assess acute changes in brain function, academic performance, working memory, inhibitory control, and sustained attention. Phase II will involve 600 children aged 10–12, randomly assigned to either a video-based PA program or a control group (300 children per group) in an 8-week cluster RCT. This phase will also incorporate a qualitative approach to explore the implementation context through pre- and post-intervention semi-structured interviews with teachers and school staff, and questionnaires for students. The outcomes of interest in this phase will include working memory, cognitive flexibility, selective attention, and academic performance. For the cross-over study, we hypothesize that PA conditions will enhance the studied outcomes compared to the control condition. In the RCT, we anticipate that the 8-week active breaks program will result in significant improvements in the selected outcomes compared to the control group. This study is expected to make pioneering contributions by including novel variables and focusing on the ADHD population. Furthermore, if the cluster RCT proves effective, it could offer a practical and cost-effective resource for integrating active breaks into daily school routines.

## 1 Background

Physical inactivity and sedentary behavior pose a global challenge, impacting not only the physical health of youth but also exerting substantial implications on their brain health (Bull et al., 2020; Guthold et al., 2020). Over the past two decades, a wealth of evidence has accumulated, indicating that physical activity (PA) is among the most promising and cost-effective strategies for improving neurocognitive function (Morales et al., 2024). Thus, promoting PA during youth development can help to enhance the likelihood of academic performance, with childhood representing a particularly crucial stage (Álvarez-Bueno et al., 2017; Lubans et al., 2016; Singh et al., 2019; de Greeff et al., 2018; Muntaner-Mas et al., 2024). Altogether, the available evidence suggests a favorable effect size (small to moderate) of both chronic and acute PA on improving various domains of educational achievement, including brain function, cognition, and academic performance (Li et al., 2017; Sibbick et al., 2022).

Researchers have examined a wide variety of educational outcomes following acute PA in children without a neurodevelopmental disorder. Specifically, some systematic reviews and metanalytic studies shed light on the field and quantified the effect sizes of acute PA. In short, results of the Watson et al. (2017) meta-analyses showed that acute PA had a positive effect on improving on-task and reducing off-task classroom behavior [standardized mean difference = 0.60 (95% CI: 0.20, 1.00)], and this fact led to improvements in academic achievement [standardized mean difference = 1.03 (95% CI: 0.22, 1.84)]. Other reviews conclude that the effects of acute PA on the working memory and cognitive flexibility domains in children are inconclusive and scarce (Pontifex et al., 2019; Paschen et al., 2019). A recent meta-analysis by de Greeff et al. (2018) reviewed studies of children, 6–12 years old, that assessed the effect of single bouts of PA on inhibition. A small to moderate positive effect of acute PA was observed (Hedges’ *g* = 0.28, 95% CI = 0.01–0.56, *p* = 0.042). Similarly, investigations of selective and sustained attention have generally observed facilitations in the ability to focus and maintain attention following acute bouts of PA in children (effect sizes ranging from 0.1 to 0.69; Pontifex et al., 2019). In conclusion, the acute effect of PA in children without a neurodevelopmental disorder seems to exert a small but positive effect on educational outcomes.

Acute PA holds also notable benefits for children with cognitive impairments like Attention Deficit Hyperactivity Disorder (ADHD), a prevalent developmental disorder affecting 5.9% of youth globally (Faraone et al., 2021). ADHD symptoms include inattention, impulsivity, and hyperactivity, leading to academic struggles and deficits in multiple cognitive domains such as working memory and inhibitory control (Harrison et al., 2019). While ADHD medication helps manage some symptoms, it does not address learning disorders and executive function deficits effectively (Faraone et al., 2015). Despite several investigations that have examined the effects of acute PA in children with ADHD the exact nature of the response on brain function, cognition, and academic performance is still uncertain. To the best of our knowledge, there is no research examining the effects of acute PA on academic performance in children with ADHD. However, acute PA have been found to elicit transient benefits for overall cognitive performance in children with ADHD (Grassmann et al., 2017; Halperin et al., 2014; Li et al., 2023; Suarez-Manzano et al., 2018; Zhu et al., 2023). Specifically, a meta-analysis suggested that acute PA significantly benefit performance on inhibition, working memory, cognitive flexibility, and attention (Pontifex et al., 2019). This primer review on the topic also concludes that exist potential moderators of effects on cognitive functions related to the specified PA parameters intensity, duration, and volume in children with ADHD. While moderate-intensity aerobic exercise has shown promising results in improving ADHD symptoms and executive functions, research on the potential effects of other PA types, durations, and intensities remains largely unknown (Chan et al., 2022; Yu et al., 2020).

Additionally, recent systematic reviews have underlined the importance of evaluating the impacts of acute PA with low and high cognitive demands on cognitive performance (Paschen et al., 2019; Skulmowski, 2024). Some authors suggest that PA with cognitive engagement incorporates added stimuli, leading to heightened physiological arousal, thereby enhancing executive functions (Tomporowski et al., 2015; Best, 2010). In this regard, PA with cognitive engagement has demonstrated superior and longer-lasting effects on executive functions and ADHD symptoms in children with ADHD (Nejati and Derakhshan, 2021). Nonetheless, findings from other studies contradict this hypothesis, suggesting that PA with cognitive engagement do not necessarily enhance cognitive performance compared to bouts of PA without cognitive engagement or sedentary activities (Bedard et al., 2021). Thus, the current evidence on the effects of PA, as short bursts, and the cognitive demands of such activity in subsequent cognitive function remains elusive.

School environments offer an ideal setting to enhance children’s educational achievement through PA, benefiting both those with and without neurodevelopmental disorders (Howie and Pate, 2012). Specifically, integrating short bursts of PA, known as active breaks, during school time has been suggested as a valuable and feasible strategy in schools settings (World Health Organization, 2024). Active breaks typically last 3–15 min and are led by teachers to enhance learning outcomes, increase daily PA and reduce sedentary time. Examples of programs are TAKE 10! (Stewart et al., 2004), Energizers (Mahar et al., 2006), Transform-US Active Break (TAB) (Lander et al., 2024), and Burn2Learn (Mavillidi et al., 2021). On the one hand, the overall results of these programs have shown promising results of active breaks on brain function, cognition, and academic performance. However, scientific reviews have yielded mixed results, attributing certain differences to the quality and design characteristics of each program (Watson et al., 2017; Daly-Smith et al., 2018; Peiris et al., 2022). Overall, while scientific progress has been made in the field, the ideal dosage of active breaks for improving educational outcomes remains uncertain.

In this context, there is limited research substantiating the adoption, implementation, and sustainability of interventions under real-world conditions (Reis et al., 2016; Wiltsey Stirman et al., 2012), such as school settings. In this scenario, implementation models, such as the Reach, Effectiveness, Adoption, Implementation, and Maintenance (RE-AIM) framework, have been devised to evaluate the public health impact of interventions (Glasgow et al., 1999). School-based PA interventions demand a comprehensive grasp of the educational system, where contextual elements—such as organizational dynamics, intervention stakeholders, and the target population—operate with greater variability compared to the controlled settings typically found in research designs (Wolfenden et al., 2022). Qualitative approaches have been justified as a valid method to develop effective interventions through the examination of facilitators and barriers by the educational agents involved (Chalkley et al., 2018; Lane et al., 2022). Nonetheless, the extant evidence indicates limited consideration of this methodology in school interventions (Gelman et al., 2023; Koorts et al., 2022).

Therefore, exploring the optimal dose of PA, particularly through classroom-based active breaks, is vital for understanding its effectiveness in children with and without ADHD. Identifying this optimal dose is crucial for maximizing the impact on academic performance, cognition, and brain function across both populations. However, a significant gap persists in the literature, especially regarding the effects of large-scale active break programs in school settings. Moreover, implementing such interventions in school settings demands a multifaceted approach, involving collaboration among teachers, school staff, and students (Daly-Smith et al., 2021).

The primary aim of this study is to present the rationale, design, and methods of the Break4Brain Project. This project will investigate: (i) the acute effects of three experimental conditions— PA with cognitive engagement, PA without cognitive engagement, and a cognitively engaging control—on academic performance, cognitive function, and brain function in children aged 10–12, both with and without ADHD, using a cross-over design in a laboratory; and (ii) the chronic effects of two experimental conditions—a video-based PA program and a control condition—on academic performance and cognitive function in school-aged children (10–12 years old) within a school setting, utilizing a cluster RCT. Additionally, it will incorporate a qualitative approach to assess the feasibility of implementing PA initiatives in school settings and will include cost-effectiveness analyses.

## 2 Methods

### 2.1 Study design

The Break4Brain project will consist of two distinct phases: phase I will employ a cross-over design (ClinicalTrials.gov identifier: NCT06303674), while phase II will utilize a hybrid type 1 implementation-effectiveness trial (ClinicalTrials.gov identifier: NCT06319833). In phase II, we will implement a type 1 hybrid trial in primary schools across the Balearic Islands (Spain; Curran et al., 2012). The primary goal of this hybrid trial is to evaluate the effectiveness of the cluster RCT. Additionally, the secondary goal aims to better understand the implementation context through a qualitative approach. The qualitative component of phase II will be developed using the most comprehensive theoretical framework available in the literature (Yoshida et al., 2020). The project has been approved by the Ethics Committee of the University of the Balearic Islands (Ref No: 301CER22 for Phase I; and Ref No: 348CER23 for Phase II). Measurement protocols of each phase have been published in Zenodo.^1^ A SPIRIT schedule and overview of the study design can be found in Tables 1A, 1B. This protocol has been developed following the Standard Protocol Items: Recommendations for Interventional Trials (SPIRIT) checklist (Supplementary File S1). This research adheres to the guidelines outlined in the Declaration of Helsinki across all its facets. Before participation, explicit written consent will be obtained from both the school staff (phase II) and parents (phase I and II), and the participants’ assent will be also sought. Any modifications to the research protocol will be transparently communicated and duly registered on ClinicalTrials.gov, ensuring accountability and compliance with ethical standards.

**Table 1A:** SPIRIT schedule of enrolment, interventions, and assessments of phase I.

	Enrolment	Allocation	Post-allocation			
Timepoint	*T1*	0	*T1 (baseline)*	*T2*	*T3*	*T4*
Enrolment						
Eligibility screen	X					
Informed consent	X					
Allocation		X				
Experimental conditions						
Cognitively engaging control				X	X	X
PA with cognitive engagement				X	X	X
PA without cognitive engagement				X	X	X
Assessments						
Brain function						
Event-related brain potentials – P3			X	X	X	X
Cognitive function						
Working memory			X	X	X	X
Inhibition			X	X	X	X
Sustained attention			X	X	X	X
Academic performance						
Academic fluency (Woodcock Muñoz^™^)			X	X	X	X
Verbal and non-verbal intelligence						
Kaufman Brief Intelligence test			X			
Physical activity patterns						
ActiGraph wGT3x-BT (21 days)			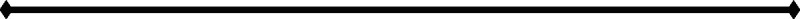			
Sedentarism patterns						
Youth Activity Profile-Spain			X			
Intensity of the active breaks						
Accelerometry (ActiGraph wGT3x-BT)				X	X	X
Heart rate monitor (Polar H10)				X	X	X
Sleep quality						
ActiGraph wGT3x-BT (21 days)			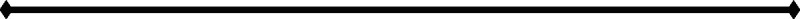			
Body composition						
Height			X			
Weight			X			
Waist circumference			X			
Hip circumference			X			
Biological maturation						
Peak height velocity			X			
Velocity of peak height growth			X			
Physical fitness						
Standing long jump test			X			
3-min step test			X			
Handgrip strenght			X			
4×10 shuttle run test			X			
Motor proficiency						
Short-form of Bruininks-Oseretsky Test of Motor Proficiency 2			X			
Mental health difficulties						
Strenght and Difficulties Questionnaire			X			
ADHD symptons						
ADHD Rating Scale-IV			X			
School grades						
Academic records			X			
Sociodemographic characteristics						
Family Affluence Scale II			X			
Parental education and occupation level						
*Ad hoc* questionnaire			X			

PA, physical activity; ADHD, Attention deficit-hyperactivity disorder.

**Table 1B:** SPIRIT schedule of enrollment, interventions, and assessments of phase II.

	Enrolment	Allocation	Post-allocation			
Timepoint	*T1*	0	T1	T2	T3	T4
Enrolment				Baseline visit	Post 8-week intervention	
Eligibility screen	X					
Informed consent	X					
Allocation		X				
Interventions						
Video-based PA program						
Control condition						
Assessments						
Design and feasibility of active breaks						
Semi-structured questionnaire (students)			X			X
Semi-structured interviews (teachers)			X			X
Semi-structured interviews (school staff)			X			X
Implementation strategies						
Professional training			X			
Direct support				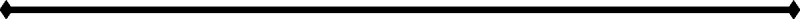		
Access to Break4Brain website				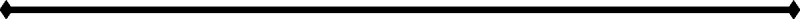		
Fidelity and follow-up of the active breaks				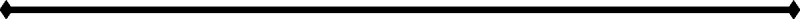		
Cognitive function						
Working memory				X	X	
Cognitive flexibility				X	X	
Selective attention				X	X	
Academic performance						
Academic fluency (Woodcock Muñoz^™^)				X	X	
Physical activity patterns						
The Physical Activity Questionnairefor Older Children (PAQ-C) a				X	X	
Sedentarism patterns						
Youth Activity Profile-Spain				X	X	
Intensity of the active breaks						
Accelerometry (ActiGraph wGT3x-BT)				X	X	
Body composition						
Height				X	X	
Weight				X	X	
Physical fitness						
International Fitness Scale				X	X	
Students classroom behavior						
Child and Adolescent Behavior Inventory				X	X	
Mental health difficulties						
Strength and Difficulties Questionnaire				X	X	
Creativity						
Adaptation of the Alternative Uses Task				X	X	
Sociodemographic characteristics						
Family Affluence Scale II				X	X	

PA, physical activity.

**Figure 1:**
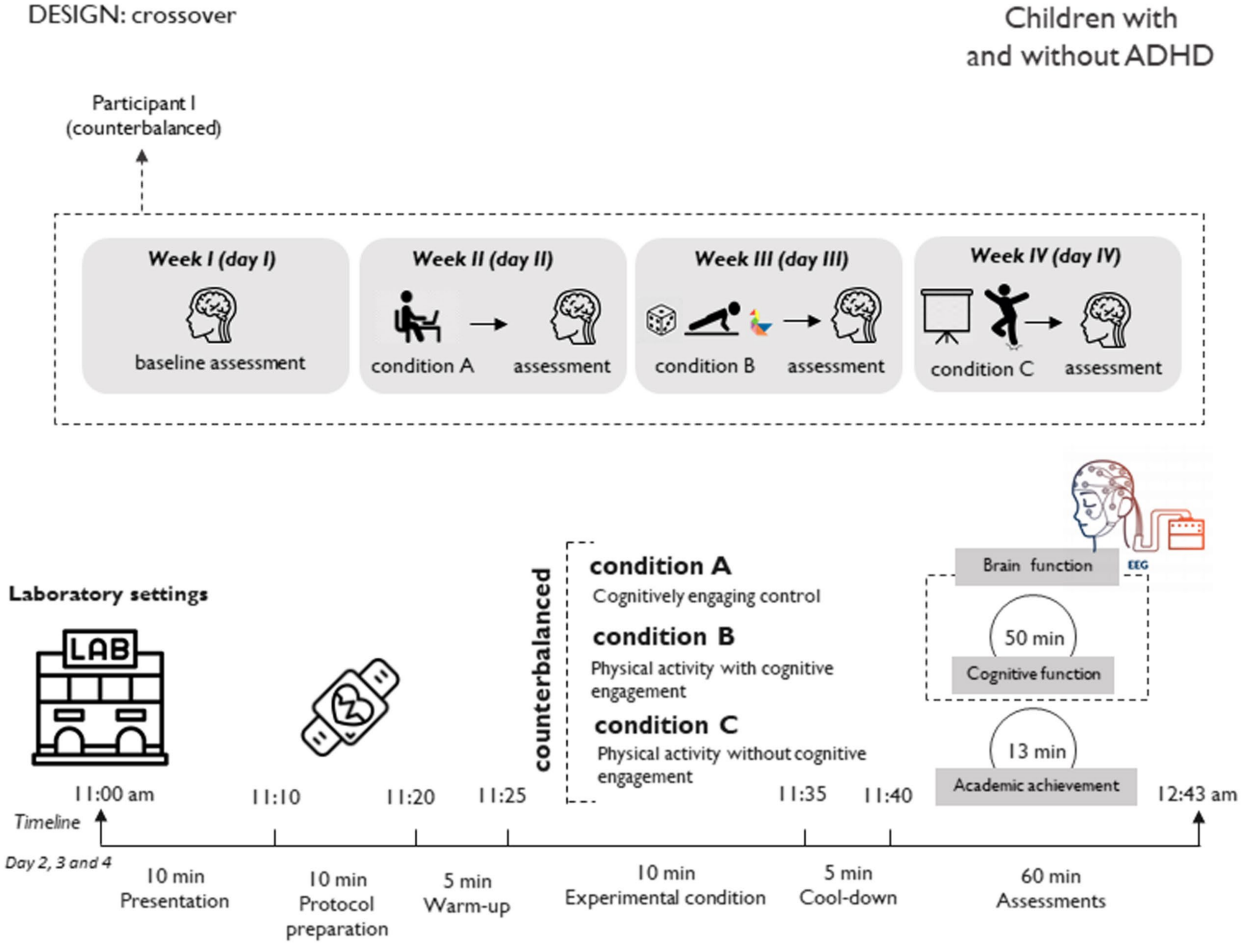
Study design and sample size for phase I (cross-over study).

In phase I (Figure 1), measurements will be conducted in laboratory settings. Each participant will undergo all experimental conditions, with the order of conditions balanced and outcomes assessed after each. Overall, participants will participate in three experimental conditions (cognitively engaging control, PA with cognitive engagement, and PA without cognitive engagement). Each participant will undergo outcome assessments four times (once at baseline, and three times post each experimental condition), with a seven-day gap between each measurement day (as well-known as washout period, enough to avoid carryover effects; Li et al., 2015). Baseline assessment will take place on the first day without engaging in any experimental conditions.

**Figure 2:**
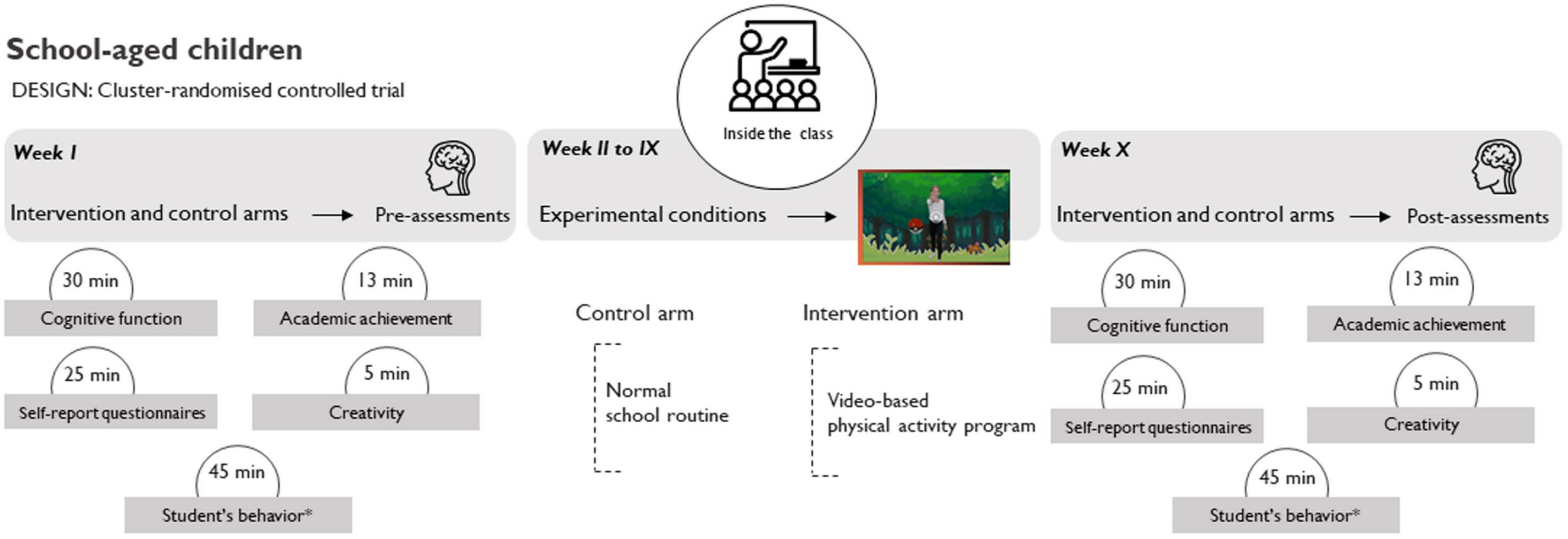
Study design for phase II (hybrid type 1 implementation-effectiveness trial: panel **(A)** - cluster randomized controlled trial; panel **(B)** - qualitative study).

Concerning phase II (Figure 2A), measurements will be carried out in school settings. Participating schools will be randomly assigned to one of two experimental conditions (control: usual academic lessons; intervention: video-based PA program [physically active without cognitive engagement]). The intervention will take 8 weeks, with the intervention condition implemented daily during this period. To design the experimental condition, a qualitative design will be conducted (Figure 2B).

### 2.2 Participants and eligibility criteria

Children aged 10–12 will be recruited for phase I through the dissemination conducted in schools, hospitals, media, and social networks across Majorca (Balearic Islands, Spain). For phase II, recruitment will be focused on children of the same age (10–12 years) from Majorca, Ibiza, and Menorca (Balearic Islands, Spain) using the same dissemination methods.

The age group selection (in both phases) will be based on late childhood’s critical brain development stage, sensitive to maturation (Giedd et al., 1999; Johnson et al., 2009). We will select preadolescents over pubertal participants to avoid the varied changes that occur during puberty, which can interefere the investigation. Our phase I focus on ADHD stems from its childhood onset and lifelong persistence, often accompanied by comorbidities (Sayal et al., 2018). Early ADHD symptom and comorbidity management is crucial, with PA suggested to alleviate adverse effects, including cognitive impairment (Halperin et al., 2014; Hoza et al., 2016). The qualitative section of phase II will involve the participation of students from the class group, teachers, and staff members of the schools. This approach will ensure feedback from the three key stakeholders essential for the successful implementation of a PA intervention in schools based on the RE-AIM framework (Yoshida et al., 2020; Kennedy et al., 2021), and complemented by Daly-Smith et al.’s guidelines (Daly-Smith et al., 2021).

The specific eligibility criteria for each phase are detailed below:

• Phase I
- (i) Children (10–12 years) diagnosed with ADHD by a psychiatrist, a psychologist or a pediatrician using the criteria of the Diagnostic and Statical Manual of Mental Disorders (DSM-5) without physical impairment; and (ii) Children (aged 10–12 years) without any neurodevelopmental or physical impairment.
• Phase II
- *Quantitative section:* (i) Children (10–12 years) without physical impairments.
- *Qualitative section:* (i) Children (10–12 years) participating in the study; (ii) Teachers of the class that will participate in the study; and (iii) School staff from a participating school.

### 2.3 Phase I: laboratory settings

#### 2.3.1 Sample size and power

A within-subjects, 3 conditions (cognitively engaging control, PA with cognitive engagement, and PA without cognitive engagement) × 4 times (baseline, posttest 1, posttest 2, and posttest 3) repeated measures ANOVA design with 15 participants should be sufficiently powered to detect interactions at or above moderate effect sizes *f* = 0.25 which translates to an approximate Cohen’s *d* = 0.5 assuming alpha at 0.05, power at 80%, and a correlation among 4 repeated measures of 0.75. Considering the primer of Pontifex et al. (2019), the median sample size utilized within the literature investigating acute effects of acute bouts of PA on cognition has been below 15 participants. Considering this together, and a potential dropout rate of 10% observed in similar studies (Mora-Gonzalez et al., 2019), our investigation will recruit 30 participants with ADHD and 30 without ADHD.

#### 2.3.2 Recruitment and randomization

Recruitment occurs out on a rolling basis, and screening procedures involve verbal screening of potential participants over the phone. Initially, the screener provides an overview of the Break4Brain Project and then obtains verbal consent. The telephone script includes questions regarding participants’ demographic information such as age, gender, and any history of ADHD, along with inquiries about inclusion and exclusion criteria. The total time administered in all screening questions is approximately 10 min. Recruitment began in January 2024 and is still ongoing. Once parents agree to participate via telephone, they sign a written informed consent before their children participate. In addition to obtaining informed consent from parents or legal guardians, this study will also seek verbal assent from the participating children. Prior to their involvement, children will be provided with age-appropriate explanations about the study’s purpose, procedures, and their role within it. They will be encouraged to ask questions and express any concerns they might have. Only those who verbally agree to participate, after understanding the study, will be included.

Randomization of participants into experimental conditions is conducted using R software by an external statistician. This randomization takes place after baseline assessments to minimize the risk of bias during measurements.

#### 2.3.3 Experimental protocol and conditions

Each participant will visit the laboratory for 4 days over 3 weeks (Figure 1). This phase comprises three experimental conditions in a counterbalanced order (Supplementary Figure S1): (i) PA with cognitive engagement, (ii) PA without cognitive engagement, and (iii) cognitively engaging control. Participants will arrive at the laboratory unaware of the experimental condition they will be practicing each day. To minimize the impact of fatigue or excessive physical exertion on performance during the experimental conditions, participants will be advised to avoid intense PA on the day prior to each visit.

##### 2.3.3.1 PA with cognitive engagement condition

This experimental condition will involve participants engaging in PA interspersed with rest periods doing cognitive tasks. Following a structured format, it consists of 10 consecutive 30-s blocks of PA alternated with cognitive tasks, totaling 10 min of PA time with a 1:1 work-to-rest ratio. Additionally, there will be 5 min allocated for warm-up and another 5 min for cool-down activities. The PA will target both aerobic and strength-based metabolism, based on the low integration-low relevance movement strategy (Mavilidi et al., 2022).

To determine the PA to be performed, participants will roll a die. Depending on the outcome, they will carry out the corresponding predetermined exercise, which was already introduced during the warm-up session. In the event of a repeat, the evaluator will replace the card for that specific exercise on the die. Conversely, during rest intervals involving cognitive tasks, participants will engage in solving various “tangram” puzzles. The researcher will manage the timing.

##### 2.3.3.2 Physical activity without cognitive engagement condition

This experimental condition will involve PA interspersed with rest periods (without doing cognitive tasks). This experimental condition will replicate the dose of the preceding one (i.e., maintaining the same work ratio, and duration). Participants will practice PA guided by video observation and imitation,^2^ based on a protocol that will not require prior experience or knowledge from the participants (Moreau et al., 2017). This video was used by the research staff in a prior study (Gelabert et al., 2023). The PA will also focus on both aerobic and strength-based metabolism, following a strategy with low integration-low relevance movements (Mavilidi et al., 2022).

##### 2.3.3.3 Cognitively engaging control condition

Participants will be instructed to watch a video concerning hygiene habits while remaining seated and at rest for 10 min.^3^ The researcher will not interact with the participants during this period. It is important to employ control conditions that closely resemble the intervention being studied. This approach helps isolate the hypothesized mechanism of interest (Simons et al., 2016). In our study, it is more clinically relevant to compare the intervention with behaviors commonly employed in classrooms, rather than solely comparing it against students sitting quietly (Pontifex et al., 2019).

#### 2.3.4 Measures

Each participant will individually attend to our laboratory on 4 different days (Figure 1), with a seven-day gap between each measurement day (once at baseline, and three times immediately after each experimental condition). Primary outcomes will be evaluated each day, while the rest will be only assessed at baseline (Table 1A). To mitigate measurement bias, trained researchers will conduct these measurements consistently in the same laboratory setting.

##### 2.3.4.1 Primary outcome measures

###### 2.3.4.1.1 Brain function

Electroencephalogram (EEG) will be recorded during both the n-back and the flanker tasks. Then, EEG datasets will be processed in Brain Vision Analyzer (Version 2.0.4), as well as through custom code implemented in MATLAB using the EEGLAB toolbox (Delorme and Makeig, 2004). From the EEG, we will obtain Event-related brain potentials (ERPs). ERPs will serve as a measure of brain response to discrete events. Electrophysiological activity will be recorded using 32 active electrodes (i.e., BrainVision actiCAP system, Brain Products GmbH, Munich, Germany). The protocol to acquire ERPs is similar to other studies examining executive functions (Galvez-Pol et al., 2018; Galvez-Pol et al., 2018; Luck, 2014). Next, we will aim to epoch the continuous data to obtain an EEG component known as P3 or P300. This component is characterized by a positive deflection in voltage around 300 milliseconds post-stimulus. It is usually elicited in tasks requiring attention and decision-making, reflecting cognitive processes related to stimulus evaluation and categorization. The P300 amplitude varies with the relevance and probability of the stimulus, serving as a key indicator of attentional and working memory processes (Luck, 2014).

##### 2.3.4.2 Secondary outcome measures

###### 2.3.4.2.1 Academic performance

The academic fluency index will be calculated, consisting of the reading, mathematics, and writing fluency subtests of the Bateria III Woodcock Muñoz^™^ (Muñoz-Sandoval et al., 2005). Reading fluency will assess the participant’s capacity to read simple sentences rapidly, mathematical fluency will gauge the ability to quickly solve simple addition, subtraction, and multiplication problems, and writing fluency will measure the ability to formulate and write sentences promptly. The median reliability coefficient alphas for all age groups for the standard battery ranged from 0.81 to 0.94.

###### 2.3.4.2.2 Cognition

A cognitive test battery covered by working memory, inhibition, and sustained attention will be administered to assess cognitive functions. All the instruments included in the study have undergone prior validation for use with the child population and cover the majority of cognitive domains affected by ADHD disorder (Wade et al., 2020).

####### 2.3.4.2.2.1 Working memory

The computerized n-back test will be utilized to evaluate the ability to update information in working memory, a component of executive control (Jacola et al., 2014). Participants will be presented with a sequence of stimuli, in this case fruits, and will need to determine if each stimulus matches the one presented ‘n’ items ago (Supplementary Figure S2). Each of the 336 trials will start with the presentation of a central fixation point, followed by an Inter-Trial Interval (ITI) lasting a maximum of 2,500 ms (varies depending on response time). Stimuli will then promptly appear after the ITI and remain on the screen for 500 ms. The n-back test will be built in Opensesame stimuli presentation software (Mathôt et al., 2012). The median reliability coefficient alpha for this task in children population ranges 0.60–0.80.

####### 2.3.4.2.2.2 Inhibition

The computerized flanker test will be used to assess the capacity to inhibit irrelevant stimuli within executive control (Krueger et al., 2009). The test is designed to measure the effect of irrelevant stimuli on cognitive processes. Specifically, participants are tasked with identifying the direction in which a central stimulus (in this case, fishes) is pointing, while simultaneously inhibiting the influence of surrounding distractor stimuli (fishes) (Supplementary Figure S3). Each of the 180 trials will begin with the presentation of a central fixation point, followed by an ITI lasting 1,100 ms, 1,300 ms, or 1,500 ms (variable time). Stimuli will then promptly appear after the ITI and will remain on the screen until the participant responds or up to 3,000 ms. The flanker test will be built in Opensesame stimuli presentation software (Mathôt et al., 2012). The Cronbach’s alpha for the flanker task in children typically falls in the range of 0.70–0.85.

####### 2.3.4.2.2.3 Sustained attention

The Conners’ Continuous Performance Test (CPT-3) will be employed to assess sustained attention (McGee et al., 2000). This test will involve presenting a sequence of 360 trials on a screen, displaying letters A through X, with each letter appearing for 250 ms. These trials will be organized into 18 consecutive blocks, each consisting of 20 trials. The intervals between stimuli will vary among the blocks, ranging from 1 to 4 s. The proportion of target stimuli (non-X letters) will remain at 90% of the total trials. The Cronbach’s alpha for the CPT-3 in children falls in the range of 0.70–0.95.

###### 2.3.4.2.3 Physical activity and sedentarism

PA and sedentarism will be measured through an objective measure such as accelerometers (Migueles et al., 2017; ActiGraph wGT3x-BT). Participants will be instructed to wear the accelerometer during their participation in the study (21 days). At baseline, a trained researcher will wear the accelerometer on the non-dominant wrist of the participant. Additionally, participants will be given a sheet where they must record when they do not have the accelerometer with them, as well as data related to sleep. They may remove the accelerometer only during water activities, including showering. Programming and data download will be done through the Actilife software (Version 6.13.4, ActiGraph, LLC). Each day that they will come to the laboratory, a trained researcher will charge the battery (once the experimental condition has been done).

###### 2.3.4.2.4 PA intensity of the experimental conditions

Intensity of the experimental conditions will be assessed using two instruments:

- Heart rate monitor. Participants will wear a Polar H10 (Speer et al., 2020) heart rate (HR) monitor connected to an iPad via the Polar Team application during the experimental conditions. This will allow us to gauge the PA intensity (classified into five resistance zones: 50–59% – 60–69% – 70–79% – 80–89% – 90–100%, of their HRmax).
- Accelerometer. The same accelerometer worn by participants since the beginning of the study will continue to be used during the execution of the experimental conditions. This device will help measure the intensity of the PA, classifying it as light, moderate, moderate-to-vigorous.

###### 2.3.4.2.5 Self-report of sedentarism

Sedentary behaviors will also be evaluated using the Youth Activity Profile—Spain (YAP-S). This self-administered questionnaire consists of 15 items and employs a 5-point Likert scale. It utilizes a 7-day recall format and has been validated for use with children (Segura-Díaz et al., 2021). In our study, participants will only need to respond to the final five questions (items 11–15), which specifically refer to sedentary habits. The Cronbach’s alpha for the YAP-S in children falls in the range of 0.70–0.85.

###### 2.3.4.2.6 Sleep quality

Sleep quality will be assessed using the previously introduced accelerometer (ActiGraph wGT3x-BT) with the same data collection procedure as reported before. Moreover, participants will maintain a sleep log, recording details such as wake-up time, daytime sleep episodes, bedtime, sleep onset latency, and any nighttime awakenings.

###### 2.3.4.2.7 Physical fitness components

Physical fitness will be evaluated using the ALPHA fitness test battery which determines speed agility by 4×10 shuttle run test, muscular fitness by handgrip strength (with a dynamometer; TKK 5101 Grip D) and standing long jump test (using a non-elastic tape; SECA 201) (Ruiz et al., 2011). Each measurement will be taken twice, and the maximum score of the two measurements will be recorded. Nonetheless, because of space limitations with our equipment, we will be unable to administer the “20-meter shuttle run test” from the ALPHA battery fitness test to assess cardiorespiratory fitness. Instead, we will adopt the validated “3-min step test” protocol (Jankowski et al., 2015). This test will involve using a step with a height of 30.5 cm with a stepping rate set at 96 beats per minute, which will be measured using a metronome. Moreover, the same pulsometer described earlier (Polar H10) will be used to determine the mean HR of one mine after the test (Speer et al., 2020).

###### 2.3.4.2.8 Motor proficiency

The short form of the Bruininks-Oseretsky Test of Motor Proficiency (BOT-2) will be used (Bruininks and Bruininks, 2005). The short form is composed of 14 subtests based on fine motor precision, fine motor integration, manual dexterity, bilateral coordination, balance, running speed and agility, upper-limb coordination, and strength. The BOT-2 has been administered in children with ADHD (Adhvaryu et al., 2022). The BOT-2 demonstrates high internal consistency, with Cronbach’s alpha values typically ranging from 0.5 to 0.99 across its various subtests and composite scores.

###### 2.3.4.2.9 Body composition

For body mass measurement, participants will be instructed to stand barefoot on an electronic scale (TANITA BC 601, Ltd) with an accuracy of 0.05 kg. Height will be measured using a stadiometer (SECA 213 Ltd) with an accuracy of 0.1 cm. Body mass index (BMI) will then be calculated as weight divided by height squared (kg/m^2^). Furthermore, waist circumference will be assessed using a non-elastic tape measure (SECA 201) with a range of 0–150 cm and an accuracy of 0.1 cm. Hip circumference will also be measured using the same non-elastic tape measure. Each measurement will be taken twice, and the mean of the two measurements will be recorded.

###### 2.3.4.2.10 Biological maturation

Using data collected, including gender, age, weight, and height, we will determine the peak height velocity (PHV; Mirwald et al., 2002). To accomplish this, the participant’s brainstem height will be measured using the same stadiometer described earlier. This measurement will be repeated twice, and the average of both will be recorded. Additionally, the length of the lower limbs will be recorded, and calculated by subtracting the brainstem height from the overall height. Furthermore, the velocity of peak height growth (VPM) will be calculated using the equations proposed by Moore et al. (2015).

###### 2.3.4.2.11 Verbal and non-verbal intelligence

The Kaufman Brief Intelligence test (K-BIT) will be used to measure verbal and non-verbal intelligence (Cordero and Calonge, 2000). All assessments are grounded in the scores attained from the vocabulary tests, which encompass 45 items of expressive vocabulary and 37 items of definitions, as well as the matrix test consisting of 48 items. This test has been used in children with ADHD (Rodriguez-Martínez et al., 2023). The KBIT- demonstrates high internal consistency, with Cronbach’s alpha values typically ranging from 0.80 to 0.96 across its verbal, non-verbal, and composite scales.

###### 2.3.4.2.12 Mental health difficulties

A parent version of the Spanish-adapted Strengths and Difficulties Questionnaire (SDQ; Barriuso-Lapresa et al., 2014) will be used to evaluate mental health difficulties in children. This questionnaire comprises 25 items and utilizes a 3-point Likert scale. It assesses five dimensions of mental health: emotional problems, behavior problems, hyperactivity-inattention, problems with peers, and prosocial behavior. Parents will provide responses based on their observations of their children’s behavior over the past 6 months. The SDQ has moderate to good internal consistency, with Cronbach’s alpha values generally ranging from 0.50 to 0.83 across its subscales and total difficulties score.

###### 2.3.4.2.13 School grades

An additional academic record will be acquired by gathering the most recent participants’ school grades. Grades in mathematics, Spanish language, Catalan language, and Physical Education will be collected, each scored on a scale from 0 to 10. Subsequently, the Grade Point Average (GPA) will be calculated as the overall average of these grades.

##### 2.3.4.3 Confounding variables

###### 2.3.4.3.1 Sociodemographic characteristics

Socioeconomic status of the children will be assessed using the Family Affluence Scale (FAS-II; Boyce et al., 2006), Version II, a validated instrument consisting of four items specifically designed for measuring socioeconomic status.

###### 2.3.4.3.2 Parental education and occupation level

Parental educational levels will be assessed using a self-report questionnaire completed by both, of the participants’ parents. The questionnaire will include options such as “no elementary school,” “elementary school,” “middle school,” “high school,” and “university completed.” Additionally, the occupations of the participants’ parents will be categorized according to the International Standard Classification of Occupations (ISCO-08) developed by the International Labor Organization. This classification comprises 13 categories: Directors and managers, Scientific and intellectual professionals, Technicians and mid-level professionals, Administrative support personnel, Service workers and salespeople in shops and markets, Farmers and skilled agricultural workers, forestry, and fishing, Officers, operators, and artisans of mechanical arts and other trades, Plant and machine operators and assemblers, Elementary occupations, Military occupations, Housemaker, and Unemployment.

###### 2.3.4.3.3 ADHD symptoms

A Spanish-adapted version of the ADHD Rating Scale-IV (Vallejo-Valdivielso et al., 2019), parent edition, will be used to gauge the frequency of symptoms linked to ADHD. This tool comprises 18 items, divided equally between inattention and hyperactivity, and employs a 3-point Likert scale. Parents will provide their assessments based on observations of their children’s behavior over the previous 6 months. The ADHD Rating Scale-IV parent edition shows excellent internal consistency, with Cronbach’s alpha values typically ranging from 0.86 to 0.96 across its subscales and total score.

#### 2.3.5 Data analysis

Analyses of the outcomes will be conducted using linear mixed models in IBM SPSS Statistics for Windows, Version 20.0 (IBM, Armonk, New York, USA), with alpha levels set at *p* < 0.05. These models will evaluate the impact of the experimental condition (PA with cognitive engagement, PA without cognitive engagement, or cognitively engaging control), time (baseline, posttest 1, posttest 2, and posttest 3), group (with and without ADHD), and condition-by-group-by-time interaction. Random effects will be incorporated to address the clustered nature of the data. Furthermore, we will assess all assumptions and adjust our analyses for any necessary control variables.

### 2.4 Phase II: school settings

#### 2.4.1 Sample size and power

A between subjects, 2 conditions (video-based PA program, and control condition) × 2 times (pretest vs. posttest) repeated measures ANOVA design with 138 participants should be sufficiently powered to detect interactions at or above moderate effect sizes *f* = 0.25 which translates to an approximate Cohen’s *d* = 0.5 assuming alpha at 0.05, power at 80%, and a correlation among 2 repeated measures of 0.75. Considering these estimations, and a potential dropout rate of 10% observed in similar studies (Mora-Gonzalez et al., 2019), our investigation will recruit 600 participants.

#### 2.4.2 Recruitment and randomization

**Figure 3:**
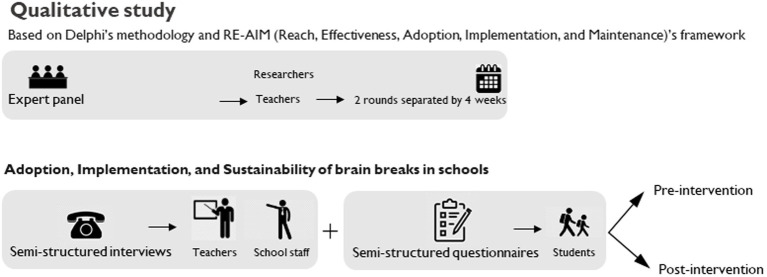
Flow diagram of the participant’s recruitment process.

Recruitment primarily involves contacting to schools in the Balearic Islands via email. The email addresses of each primary school were acquired from the Spanish Ministry of Education website. Once recruited, an initial virtual meeting was made to explain the study’s objectives. Figure 3 illustrates the flow diagram of the participant’s recruitment process. Recruitment began in March 2024 and is still ongoing. Once teachers and parents agree to participate, they sign a written informed consent form before the start of the intervention. In line with phase I, this study will also obtain verbal assent from participating children. Only those who provide verbal agreement, having fully understood the study’s aims and procedures, will be included.

Randomization of schools into experimental conditions for phase II is carried out using R software by an external statistician.

#### 2.4.3 Experimental protocol

The experimental protocol will be composed of two parts: (a) qualitative: the experimental condition will be developed through a qualitative design involving semi-structured interviews with teachers and school staff, along with semi-structured questionnaires administered to students (Figure 2B). The same process will be replicated after the intervention; (b) quantitative: the intervention will be implemented within school settings during regular curricular hours, taking place inside the classroom (Figure 2A). Specifically, schools randomly selected and assigned to the research arms (video-based PA program, or control condition) will conduct educational achievement assessments twice, separated by 8 weeks. The initial assessment will occur at the pretest stage (week 1), followed by a second assessment immediately after 8 weeks of intervention (week 10).

##### 2.4.3.1 Video-based physical activity program

The experimental condition will occur daily within regular academic lessons. Students in each classroom will engage in the intervention once a day. The classroom teacher, responsible for conducting the intervention, will choose the appropriate time for implementation. The intervention will not be scheduled during PE lessons or immediately after them, nor during lessons following recess.

The administration of this intervention will use a digital platform accessible to each classroom teacher via a password-protected link.^4^ This platform will facilitate the easy and efficient administration of the video-based PA program. It will feature 10 videos, with five different themes, administered in a randomized manner according to the researcher’s guidelines. However, no theme will repeat throughout the week. To ensure proper use of this tool, classroom teachers will undergo training conducted by the research team before initiating the intervention.

During the intervention, students will follow the instructions in the video and replicate the PA in a gamified format. The structure of the video-based PA program will comprise seven sets of 40-s PA alternated with seven sets of 20-s rest periods, totaling 7 min of intervention with a 2:1 work-to-rest ratio. The PA will target both aerobic and strength-based metabolism, following a strategy with low integration-low relevance movements (Mavilidi et al., 2022).

##### 2.4.3.2 Control group

During the 8-week intervention period, the control group will continue receiving usual academic lessons, without the inclusion of PA that may impact their typical levels during school hours.

#### 2.4.4 Measures

Each educational agent (teachers, school staff, and students) will be involved in the qualitative aspect of this phase and will undergo evaluation 1 month before and 1 month after the intervention. On the other hand, each student from each participating school will undergo evaluation for quantitative variables both before the intervention (1 week before its start) and after the intervention (1 week following its conclusion).

##### 2.4.4.1 Primary outcome measures

###### 2.4.4.1.1 Cognition

A cognitive test battery encompassing working memory, cognitive flexibility, and selective attention will be administered to evaluate cognitive functions. All tests used in this study have undergone previous validation for their appropriateness with the children population.

###### 2.4.4.1.2 Working memory

An adapted version of the digit memory test will be used to measure the working memory (Hill et al., 2010). Students will listen to digit sequences and write them down (i.e., 1, 4, 6). If successful, longer sequences will be given until two consecutive failures occur. One researcher dictates while another oversees. This test includes: “Digits Forward,” where participants write the digits in the given order, and “Backward Digit-Span,” where participants write the digits in reverse order.

###### 2.4.4.1.3 Cognitive flexibility

An adapted version of the Trail-making test (TMT) will be used to assess cognitive flexibility (Howie et al., 2015). The TMT will comprise two parts: (a) TMT-A will evaluate processing speed and visual attention, requiring participants to connect numbers from 1 to 25 in order; (b) TMT-B will assess cognitive flexibility by alternating between numbers and letters in ascending and alphabetical order. Students will record completion times using a digital stopwatch displayed on a screen. The test will be supervised by the two researchers. For the Trail Making Test (TMT) used with children, Cronbach’s alpha generally falls within 0.60–0.80 for its subtests, reflecting moderate reliability.

####### 2.4.4.1.3.1 Selective attention

The d2, test of attention will be used to assess the selective attention (Brickenkamp, 2012). Students must carefully scan each of the 14 lines of the test from left to right, identifying any “d” with two small lines. These are considered relevant elements, while other combinations are irrelevant. Each line allows 20 s for assessment, with one researcher monitoring time and another ensuring the process runs smoothly. For the d2 in children, Cronbach’s alpha values are generally reported to be between 0.75 and 0.90, indicating strong internal consistency.

##### 2.4.4.2 Secondary outcome measures

###### 2.4.4.2.1 Academic performance

The academic fluency index and their subtests will be assessed similarly to phase I of the study (Muñoz-Sandoval et al., 2005). However, the procedure will vary. In this instance, all students within a specific classroom will undertake the tests simultaneously, with two trained researchers overseeing to ensure that each student completes it independently (time will be monitored using a chronometer).

###### 2.4.4.2.2 Design and feasibility of active breaks

The responses collected from semi-structured interviews with teachers and school staff, along with the semi-structured questionnaires administered to students, will be instrumental in shaping the design and assessing the feasibility of implementing active breaks. These tools will be rigorously validated through a Delphi methodology, involving a panel of at least 10 experts (Gillis et al., 2013). The questions will be developed using the RE-AIM framework and further refined according to the guidelines provided by Daly-Smith et al. (2021).

###### 2.4.4.2.3 Physical activity and sedentarism

PA and sedentarism will be calculated by accelerometry (ActiGraph wGT3x-BT), using the same instrument and procedure in phase I (Migueles et al., 2017). Due to the limited availability of accelerometers, one accelerometer will be assigned to each classroom. This accelerometer will rotate among students daily (24-h) until each student has had a turn, being the classroom teacher responsible for its safekeeping. The classroom teacher will keep track of who will wear the accelerometer every day. Moreover, a researcher will handle data retrieval and recharge the battery once a week.

###### 2.4.4.2.4 Intensity of the active breaks

Intensity of the active breaks will be measured by accelerometry (Migueles et al., 2017). Following the procedure of the above section, intensity of the active breaks will be measured. For this reason, the classroom teacher will receive a sheet to note at which time (hours: minutes: seconds) started and finished every active break.

###### 2.4.4.2.5 Self-report of physical activity patterns

An adapted Spanish version of the PA questionnaire for children (PAQ-C) will be administered to evaluate PA patterns (Manchola-González et al., 2017). This self-administered questionnaire employs a seven-day recall period, comprising nine items measured on a 5-point Likert scale. For the PAQ-C, Cronbach’s alpha generally ranges from 0.70 to 0.85, reflecting good internal consistency.

###### 2.4.4.2.6 Self-report of sedentarism

Sedentary patterns will be calculated using the same instrument and procedure (although, in this case, the evaluation will take place within a classroom setting) as in phase I (Segura-Díaz et al., 2021).

###### 2.4.4.2.7 Sleep quality

Following the established procedure for measuring PA and sedentary patterns, sleep quality will be assessed using accelerometry (ActiGraph wGT3x-BT).

###### 2.4.4.2.8 Self-report of physical fitness

Physical fitness will be measured through the adapted Spanish version of the International Fitness Scale (Sánchez-López et al., 2015). This instrument is composed of five items on a 5-point Likert scale. The Cronbach’s alpha of the International Fitness Scale in children, is generally reported to be between 0.70 and 0.85, indicating good internal consistency.

###### 2.4.4.2.9 Self-report of body composition

The height (cm) and weight (kg) of the students will be self-reported by parents, which is a reliable alternative to direct measurements (Rios-Leyvraz et al., 2022). Then, BMI will be also calculated (kg/m^2^).

###### 2.4.4.2.10 Students’ classroom behavior

Classroom teachers will employ two parts (inattention and hyperactivity-impulsivity) of the Child and Adolescent Behavior Inventory (CABI). Each of these parts is composed of nine items and uses a 6-point Likert scale (Burns et al., 2023).

###### 2.4.4.2.11 Creativity

An adaptation of the Alternative uses task (AUT) will be used to measure creativity (Olson et al., 2021). Students will be required to complete the maximum number of unusual uses for two objects within a 3-min timeframe (with one researcher overseeing the timing and another guaranteeing of the process).

##### 2.4.4.3 Confounding variables

The socioeconomic status of the children will be assessed using the validated Family Affluence Scale (FAS-II), which consists of four items specifically designed to measure socioeconomic status (Boyce et al., 2006). Additionally, self-reported body composition and self-reported PA patterns will also be considered as confounding variables in the analysis.

#### 2.4.5 Implementation strategies and cost-effectiveness analyses

To guarantee the correct development of the intervention, a few implementation strategies will be conducted during the intervention period providing professional training for teachers (i.e., initial meeting before the start of the intervention); offering direct support from researchers (i.e., weekly visit at the school, and direct contact by e-mail or WhatsApp); granting access to the Break4Brain website for administering active breaks; and ensuring fidelity and follow-up of the active breaks (i.e., recording one active break weekly, and documenting daily participation of students).

Concerning the cost-effectiveness analyses, trial costs, and outcomes will be extrapolated to the Spanish population aged 10–12 in government schools, to assess the potential lifetime health and cost outcomes resulting from the intervention (Brown et al., 2024). The cost-effectiveness model will be determined using the validated ACE-Obesity Policy model (Ananthapavan et al., 2020).

#### 2.4.6 Data analysis

##### 2.4.6.1 Quantitative analysis

The outcomes will be analyzed utilizing linear mixed models in IBM SPSS Statistics for Windows, V.20.0 (IBM, Armonk, New York, USA), with significance levels set at *p* < 0.05. These models will evaluate the effects of the condition (video-based PA program and control group), time (pre and posttest), and the interaction between condition and time. Random effects will be incorporated to address the nested nature of the data, adjusting for clustering effects at the school level. Additionally, baseline PA levels, obesity status, gender, and age will be considered as potential moderators in the analysis. Moreover, all assumptions will be tested, and analyses will be adjusted for control variables if necessary.

##### 2.4.6.2 Qualitative analysis

Qualitative data will be gathered through semi-structured interviews and questionnaires administered by trained researchers. Thematic analysis techniques and a grounded theory approach will organize and manage all essential information obtained from the interviews. Quantitative data of the semi-structured questionnaire will be summarized into means and standard deviations. Semi-structured interviews will be audio-recorded, transcribed verbatim, and anonymized before coding. The analysis will be conducted using “NVivo version 12 Plus” software (QRS International-Melbourne, Australia).

## 3 Discussion

The main objective of the Break4Brain Project is to examine the acute and chronic effects of PA (either with or without cognitive engagement) on academic performance, cognitive function, and brain function in ADHD-diagnosed and non-diagnosed children, in controlled laboratory settings and schools.

Recently, there has been a surge in studies evaluating the impact of active breaks on various educational outcomes in children (Watson et al., 2017; Daly-Smith et al., 2018). A predominant focus of these studies has been on investigating the effects of PA specifically on children without neurodevelopmental disorders. A strength of this study is that it will compare the findings of children with and without ADHD. Thus, an exploration into this area is warranted to elucidate potential variations in response to acute PA, ultimately facilitating the development of more tailored and effective interventions (in parameters of duration, intensity, or PA type) in both populations.

Based on previous research, our hypothesis for the cross-over design (phase I) is that the experimental conditions involving PA will enhance performance on the studied outcomes. Our recent meta-analysis found that a single bout of PA significantly improves academic outcomes Hedge’s *g* = 0.35, 95% CI: 0.20–0.50; Muntaner-Mas et al., 2024. However, while our meta-analysis highlighted the overall benefits of PA, it did not reveal that specific characteristics such as intensity or duration significantly moderated the magnitude of these effects. This absence of moderation raises intriguing questions about what other factors might drive the observed improvements. We speculate that the type of PA, rather than just its intensity or duration, may play a crucial role in influencing outcomes. Specifically, we draw on Mavilidis’ theory-driven approach (Mavilidi et al., 2022), which emphasizes the importance of the cognitive and contextual relevance of PA in enhancing learning and academic achievement. According to this framework, physical activities that are less integrated with academic content and have lower cognitive demands may paradoxically yield more significant improvements in certain outcomes. Thus, we speculate that our experimental conditions characterized by low integration and low relevance may lead to the most substantial gains in the studied outcomes. By exploring these nuanced interactions between PA type and academic achievement, our study aims to advance the understanding of how different forms of PA can be strategically employed to enhance educational outcomes, particularly in settings involving children with varying cognitive needs and abilities.

Despite numerous investigations into the effects of PA in children with ADHD, the precise impact on brain function, cognition, and academic performance remains elusive (Chan et al., 2022; Welsch et al., 2021). Current research has primarily focused on specific outcomes or short-term effects, often neglecting a holistic approach that includes a comprehensive battery of tests to assess these variables simultaneously. To the best of our knowledge, there is a significant gap in research that thoroughly examines the effects of acute PA on both brain function and academic performance in children with ADHD, particularly in comparison to their non-ADHD peers. This gap is especially critical given that children with ADHD may exhibit unique responses to PA interventions due to their distinct neurocognitive and behavioral characteristics. These differences suggest that PA might influence ADHD and non-ADHD children in divergent ways, potentially offering specialized benefits or necessitating tailored intervention strategies to maximize effectiveness. For example, a meta-analysis by Li et al. (2023) found that cognitively engaging exercises are particularly effective in improving attention problems in school-aged children with ADHD. This highlights the possibility that specific types of PA, especially those that engage cognitive processes, could be more beneficial for children with ADHD compared to their non-ADHD counterparts.

Regarding school settings, there has often been a disconnect between research evidence and the practical implementation of past interventions (McMullen et al., 2014). For instance, while existing evidence suggests certain strategies might be effective, qualitative feedback from educators reveals differing preferences. Teachers, for example, have consistently expressed a preference for short, video-based PA breaks lasting less than 10 min. This highlights the importance of aligning research with the practical realities and preferences within educational settings to enhance both feasibility and effectiveness. To address this challenge, our cluster RCT will be collaboratively designed with input from educational stakeholders, ensuring that the strategies are not only evidence-based but also practical, sustainable, and aligned with the day-to-day demands of teachers and students. This collaborative approach is crucial for the successful integration of active breaks into the school routine, as it fosters a sense of ownership and relevance among educators, which in turn, increases the likelihood of long-term adoption and sustainability. Moreover, the Break4Brain project’s digital platform significantly alleviates the workload for teachers by providing pre-designed, video-based PA breaks. These videos include clear, step-by-step instructions, allowing teachers to easily implement the activity by simply selecting the appropriate video. This system minimizes the preparation required by teachers and seamlessly integrates PA into the classroom environment without disrupting the flow of the school day. In this sense, and to our knowledge, this is the most comprehensive study in the field of active breaks, as it not only incorporates rigorous research methodologies but also addresses the practical needs of educators through a hybrid type 1 implementation-effectiveness trial. This approach ensures that the interventions are both effective in improving student outcomes and feasible for long-term implementation in real-world educational settings, bridging the gap between research and practice in a meaningful way.

Another strength of this study is that it will measure the fidelity of each student (and class) to the intervention. A recent systematic review and meta-analysis revealed that most published studies have failed to assess fidelity to active breaks (Ruhland and Lange, 2021). This underscores the significance of our approach in ensuring that interventions are implemented as intended, thereby enhancing the reliability and validity of our findings. Additionally, following statements from other systematics reviews (Watson et al., 2017; Infantes-Paniagua et al., 2021), PA patterns and active break intensity will be measured with objective instruments such as accelerometers or HR monitors (in both phases). On the other hand, several limitations warrant mention: (i) the generalizability of the results may be limited to our population context (encompassing factors such as primary school stage, socioeconomic status, etc.); (ii) resource constraints will necessitate the daily rotation of accelerometers among students during phase II.

## Footnotes

^1^ https://doi.org/10.5281/zenodo.8374014

^2^ https://www.youtube.com/watch?v=PN7w1X5jPK0

^3^ https://www.youtube.com/watch?v=rZr8geozI_s&list=LL&index=10

^4^ https://www.opospills.com/break4brain
